# Progress in Ophthalmology

**Published:** 1895-08-17

**Authors:** 


### PROGRESS IN OPHTHALMOLOGY.

(*Continued from page 292.*)

**Infantile Glaucoma producing Buphthalmos.**—Dr. Snelling, of Utrecht,[1] speaks favourably of repeated paracentesis of the anterior chamber in this affection, combined with the energetic use of myotics. He makes his punctures at the limbus, and repeats them every three days, apparently for a considerable period of time.

**Tenotomy of the Superior or Inferior Recti in Strabismus.**—Dr. Stevens,[2] of New York, devotes two papers to the consideration of cases in which convergent strabismus is due to want of equilibrium between the superior and inferior recti, and is the result of an effort to correct a tendency to upward or downward displacement of one or both eyes. Such cases, according to him, are not uncommon, and are often attended by a high degree of asthenopia. They require tenotomy of the muscles primarily affected, and not of the lateral recti; and the author asserts that tenotomies which are insufficient completely to correct the faulty position will nevertheless prove highly valuable in diminishing the asthenopia. The cases require very careful examination before any operation for their relief can be profitably undertaken. Dr. Stevens promises another paper, upon cases in which defective equilibrium of the ocular muscles, although not producing manifest strabismus, is nevertheless a cause of asthenopia, and sometimes of general symptoms indicative of nervous exhaustion.

**Diphtheritic Conjunctivitis Treated by Antitoxine.**—Dr. Morax[3] relates four cases, in all of which the bacillus of diphtheria was present, and which all recovered without injury to the eyes. The only local treatment was by washing with warm water and with a solution of boric acid, and the serum was administered in doses of from 10 to 30 c.c., according to severity and to the age of the patient. In two of the cases single doses of 10 c.c. were sufficient.

**Posterior Sclerotomy and Sclerectomy in Glaucoma.**—Dr. Parinaud[4] advocates these proceedings, more especially in the chronic forms of glaucoma. By "posterior sclerotomy" he means a simple puncture, made with a Graefe's knife not less than two millimetres in width, at a point about 8 mm. distant from the corneal margin. The conjunctiva being first drawn aside, the knife is pushed from four to six mm. into the eyeball, in a direction towards its centre, is turned slowly in a quarter of a circle, and withdrawn, the conjunctiva then being suffered to return into its natural position, so that the external wound will not coincide with that in the sclera. To perform "sclerectomy" he draws aside the conjunctiva as before, raises the superficial portion of the sclera on a fine needle, and then cuts off the raised portion in such a way as to leave a saucer-like depression in the membrane. Either at the same time, or, more frequently, after the lapse of three or four days, the eye is punctured with a needle at the lowest point of the depression. In both operations, the best results are obtained when there is a complete absence of inflammatory reaction, and this result is said to be best secured when care is taken not to make the puncture or the excision too large. No precise figures or statistics are given in support of the statements in the paper. In the following number of the *Annales* (Juin, '95) Dr. Nicati claims to have been the originator of posterior sclerotomy, and asserts that it is never of permanent efficacy.

**Traumatic Hernia of the Lachrymal Gland.**—Dr. Haltenhoff[5] relates the case of a child, two and a-half years old, who fell face downwards upon a road covered with broken stone, and who received an incised wound of the upper lid, through which the lachrymal gland protruded. After three days it was excised, and a good recovery followed. The author has been able to find only three recorded cases of a similar accident, in one of which the late Albrecht v. Graefe succeeded in reducing the protrusion.

[1] Ann [illegible]'Ocul., March, 1895. [2] Ann. d'Ocul., April and June, 1895. [3] Ann. [illegible]l., April, 1895. [4] Ann. d'Ocul., May, 1895. [5] Ann. d'Ocul., Mar[illegible]

